# Metastasis pattern and prognosis of large cell neuroendocrine carcinoma: a population-based study

**DOI:** 10.1007/s00432-023-04975-w

**Published:** 2023-07-27

**Authors:** Tongchao Jiang, Haishuang Sun, Na Li, Tongcui Jiang

**Affiliations:** 1 Department of Radiotherapy, State Key Laboratory of Oncology in South China, Guangdong Key Laboratory of Nasopharyngeal Carcinoma Diagnosis and Therapy, Collaborative Innovation Center for Cancer Medicine, Sun Yat-Sen University Cancer Center, Guangzhou, 510060 Guangdong China; 2 Department of Medical Oncology, Sun Yat-Sen University Cancer Center; State Key Laboratory of Oncology in South China, Collaborative Innovation Center for Cancer Medicine, Guangzhou, 510060 Guangdong China; 3 Division of Life Sciences and Medicine, Department of Neurosurgery, The First Affiliated Hospital of USTC, University of Science and Technology of China, 81 Meishan Road, Shushan District, Hefei, 230000 Anhui China; 4 School of Basic Medical Sciences, Anhui Medical University, Hefei, 230032 Anhui China; 5 Biopharmaceutical Research Institute, Anhui Medical University, Hefei, 230032 Anhui China; 6 Yinghua Dong Street, Hepingli, Chao Yang District, Beijing, 100029 China

**Keywords:** Large cell neuroendocrine carcinoma (LCNEC), Metastasis, SEER database, Nomogram, Prognostic model

## Abstract

**Purpose:**

As a rare type of tumor, the metastasis pattern of large cell neuroendocrine carcinoma (LCNEC) is still unclear. Our aim was to investigate metastatic patterns and develop a predictive model of prognosis in patients with advanced LCNEC.

**Methods:**

Patients of LCNEC diagnosed between 2010–2015 from the Surveillance, Epidemiology and End Results (SEER) database were retrospectively included. Chi-square test was used for baseline characteristics analysis. Survival differences were assessed using Kaplan–Meier curves. Independent prognostic factors identified by multivariate Cox proportional risk model were used for the construction of nomogram.

**Results:**

557 eligible patients with metastasis LCNEC (median (IQR), 64 (56 to 72) years; 323 males) were included in this research. Among patients with isolated metastases, brain metastases had the highest incidence (29.4%), and multisite metastases had worse OS (HR: 2.020: 95% CI 1.413–2.888; *P* < 0.001) and LCSS (HR: 2.144, 95% CI 1.480–3.104; *P* < 0.001) in all age groups. Independent prognostic indicators including age, race, T stage, N stage, chemotherapy, radiotherapy and metastatic site were used for the construction of nomogram. Concordance index (C-index) and decision-curve analyses (DCAs) showed higher accuracy and net clinical benefit of nomogram compared to the 7th TNM staging system (OS: 0.692 vs 0.555; *P* < 0.001; LCSS: 0.693 vs 0.555; *P* < 0.001).

**Conclusions:**

We firstly established a novel comprehensive nomogram to predict the prognosis of metastasis LCNEC. The prognostic model demonstrated excellent accuracy and predictive performance. Chemotherapy and metastasis pattern were the two strongest predictive variables. Close follow-up of patients with LCNEC is necessary to make individualized treatment decisions according to different metastasis patterns.

**Supplementary Information:**

The online version contains supplementary material available at 10.1007/s00432-023-04975-w.

## Introduction

Pulmonary large-cell neuroendocrine carcinoma (LCNEC) originates from endocrine cells of lung and bronchial epithelium and is a type of rare tumor that accounts for 3% of lung cancers (Fasano et al. 2015; Rekhtman 2010). In 2015, the World Health Organization (WHO) classified it as neuroendocrine tumor along with typical carcinoid, atypical carcinoid and small-cell lung cancer (SCLC) (Travis et al. 2015). LCNEC and SCLC share many similar clinicopathological features and are categorized as high-grade neuroendocrine carcinoma, which is characterized by highly aggressiveness, poor histologic differentiation, poor prognosis, susceptibility to metastasis and often at advanced stage at the time of diagnosis (Stamatis 2014; Ferlay et al. 2015). The most common metastasis site is the lung, however, some patients involve not only one organ, and the prognosis often varies across metastasis sites (Chen et al. 2020). Although new therapeutic strategies such as targeted therapy have improved the disease process, advanced lung cancer remains an incurable disease (Reckamp et al. 2020). Therefore, it is particularly important to clarify the prognostic factors of metastatic lung cancer for treatment decision making. In recent years, several studies have reported prognostic risk factors and developed prognostic models for LCNEC (Kinslow et al. 2020; Ma et al. 2021; May et al. 2021).However, to our knowledge, large-scale studies on metastatic LCNEC have not been reported. In this research, we firstly investigated the metastatic pattern and established comprehensive nomograms to assess 1-, 2-, and 3-years OS and LCSS of advanced LCNEC based on data from the Surveillance, Epidemiology, and End Results (SEER) database.

## Materials and methods

### Ethical approval

As all data were obtained from the SEER database, informed patient consent and ethical approval were not required.

### Data source

The data analyzed in the study were obtained from the SEER database, which covered almost 30% of the entire U.S. population. Information is provided including cancer incidence, treatment options, staging and survival. SEER*Stat 8.3.5 software was performed (http://seer.cancer.gov/SEERSTAT/) to access the database.

### Patient selection

Since metastatic site codes were available from 2010 in the SEER database, patients diagnosed with LCNEC between 2010 and 2015 were enrolled in this study to ensure adequate follow-up time. The inclusion criteria for all patients were as follows: (1) located in the lung and bronchus; (3) histological diagnosis of LCNEC (histologic codes 8013/3); (4) patients with 7th American Joint Committee on Cancer (AJCC) Stage IV. Patients excluded from our study were as follows: (1) the unknown TNM stage; (2) the unknown distant metastasis information; (3) follow-up information was incomplete; (4) patients with multiple primary sites. Endpoints included overall survival (OS) and lung cancer-specific survival (LCSS). OS was the time from diagnosis to death from all causes or the last follow-up. And LCSS was the survival time from the date of diagnosis to a specific cancer-related death.

### Statistical analyses

Differences between groups were assessed by chi-square test. Kaplan–Meier method was used for survival analysis, and differences between curves were tested by log-rank test. Risk factors of OS and LCSS were determined by univariate and multivariate Cox regression models. Prognostic factors with *P* < 0.05 in multivariate Cox regression analysis were applied to construct nomograms to predict survival outcomes. The discriminability of the model was assessed by calculating the Harrell’s concordance index (C-index) with a 95% confidence interval (CI). Calibration curves was applied to compare the predicted probabilities between actual survival and the nomograms. Eventually, evaluating the net benefit and potential clinical utility of the model via decision-curve analyses (DCAs). The ability of the model based on the 7th TNM staging and the nomograms established in our research was compared with the use of C-index and DCAs. All statistical analyses were performed with SPSS statistical analysis software (version 24.0, IBM Corporation, Armonk, NY, USA) and R (version 3.6.0, R Foundation for Statistical Computing, Vienna, Austria). *P*-values were bilateral, the result with *P* < 0.05 was defined as a statistically significant.

## Results

### Patient characteristics

A total of 557 LCNEC patients with definite metastatic sites were included in this study. The detailed flow chart of the research was shown in Figure S1. The median age of the total population was 64 years (interquartile range (IQR), 56–72). The patients were mostly male (*n* = 323, 58%) and most frequently in whites (*n* = 472, 84.7%). Additionally, the primary tumor of LCNEC was more likely to occur in the right lung (*n* = 264, 47.4%), and predominantly in the upper lobe (*n* = 312, 56%). Detailed demographic characteristics were described in Table 1.

**Table 1:** Patients’ demographics and clinicopathological characteristics of different metastasis patterns

Clinical features	Bone only	Brain only	Liver only	Lung	Multiple	*P* value
	*n* = 73 (13.1%)	*n* = 164 (29.4%)	*n* = 72 (12.9%)	*n* = 42 (7.5%)	*N* = 206 (37%)	
**Age**						**0.010**
< 65	56 (63.0)	87 (53.0)	26 (36.1)	17 (40.5)	110 (53.4)	
≥ 65	27 (37.0)	77 (47.0)	46 (63.9)	25 (59.5)	96 (46.6)	
**Sex**						**0.022**
Female	22 (30.1)	84 (51.2)	32 (44.4)	15 (35.7)	81 (39.3)	
Male	51 (69.9)	80 (48.8)	40 (55.6)	27 (64.3)	125 (60.7)	
**Race**						0.448
White	58 (79.5)	138 (29.2)	64 (13.6)	33 (7.0)	179 (37.9)	
Black	11 (15.1)	22 (33.8)	5 (7.7)	8 (12.3)	19 (29.2)	
Other	4 (5.5)	4 (20.0)	3 (15.0)	1 (5.0)	8 (40.0)	
**Marital status**						0.202
Married	47 (64.4)	93 (56.7)	37 (51.4)	16 (38.1)	104 (50.5)	
Unmarried	24 (32.9)	65 (39.6)	34 (47.2)	25 (59.5)	97 (47.1)	
Unknown	2 (2.7)	6 (3.7)	1 (1.4)	1 (2.4)	5 (2.4)	
**Laterality**						0.198
Left	22 (30.1)	60 (36.6)	23 (31.9)	22 (52.4)	79 (38.3)	
Right	46 (63.0)	87 (53.0)	44 (61.1)	19 (45.2)	116 (56.3)	
Others	5 (6.8)	17 (10.4)	5 (6.9)	1 (2.4)	11 (5.3)	
**Primary location**						0.244
Upper lobe, lung	41 (56.2)	83 (50.6)	32 (44.4)	18 (42.9)	90 (43.7)	
Middle lobe, lung	2 (2.7)	5 (3.0)	8 (11.1)	0 (0)	11 (5.3)	
Lower lobe, lung	14 (19.2)	39 (28.3)	14 (19.4)	14 (33.3)	53 (25.7)	
Main bronchus	5 (6.8)	7 (4.3)	9 (12.5)	4 (9.5)	19 (9.2)	
Overlapping lesion of lung	1 (1.4)	1 (0.6)	1 (1.4)	0 (0)	1 (0.5)	
Lung, NOS	10 (13.7)	29 (17.7)	8 (11.1)	6 (14.3)	32 (15.3)	
**Grade**						0.064
I–III	23 (31.5)	36 (22)	16 (22.2)	6 (14.3)	48 (23.3)	
IV	9 (12.3)	18 (11)	2 (2.8)	6 (14.3)	12 (5.8)	
Unknown	41 (56.2)	110 (67.1)	54 (75)	30 (71.4)	146 (70.9)	
**T stage**						**0.003**
T0–1	14 (19.2)	32 (19.5)	8 (11.1)	3 (7.1)	17 (8.3)	
T2	14 (19.2)	40 (24.4)	23 (31.9)	8 (19)	52 (25.2)	
T3	14 (19.2)	34 (20.7)	12 (16.7)	9 (21.4)	48 (23.3)	
T4	25 (34.2)	34 (20.7)	16 (22.2)	21 (50)	66 (32)	
Tx	6 (8.2)	24 (14.6)	13 (18.1)	1 (2.4)	23 (11.2)	
**N stage**						**< 0.001**
N0	13 (17.8)	63 (38.4)	13 (18.1)	10 (23.8)	25 (12.1)	
N1	6 (8.2)	17 (10.4)	7 (9.7)	2 (4.8)	21 (10.2)	
N2	30 (41.1)	62 (37.8)	36 (50)	20 (47.6)	97 (47.1)	
N3	20 (27.4)	17 (10.4)	13 (18.1)	10 (23.8)	54 (26.2)	
Nx	4 (5.5)	5 (3)	3 (4.2)	0 (0)	9 (4.4)	
**Surgery**						
No/unknown	71 (97.3)	149 (90.9)	70 (97.2)	38 (90.5)	200 (97.1)	**< 0.001**
Yes	2 (2.7)	15 (9.1)	2 (2.8)	4 (9.5)	6 (2.9)	
**Radiation**						**< 0.001**
No	54 (74.0)	84 (51.2)	68 (94.4)	38 (90.5)	171 (83.0)	
Yes	19 (26.0)	80 (48.8)	4 (5.6)	4 (9.5)	35 (17.0)	
**Chemotherapy**						0.124
No/unknown	19 (26.0)	16 (39.0)	25 (34.7)	11 (26.2)	58 (28.2)	
Yes	50 (74.0)	100 (61.0)	47 (65.3)	31 (73.8)	148 (71.8)	

*P* values in bold signify a statistically significant difference

### Metastasis patterns

**Fig. 1:**
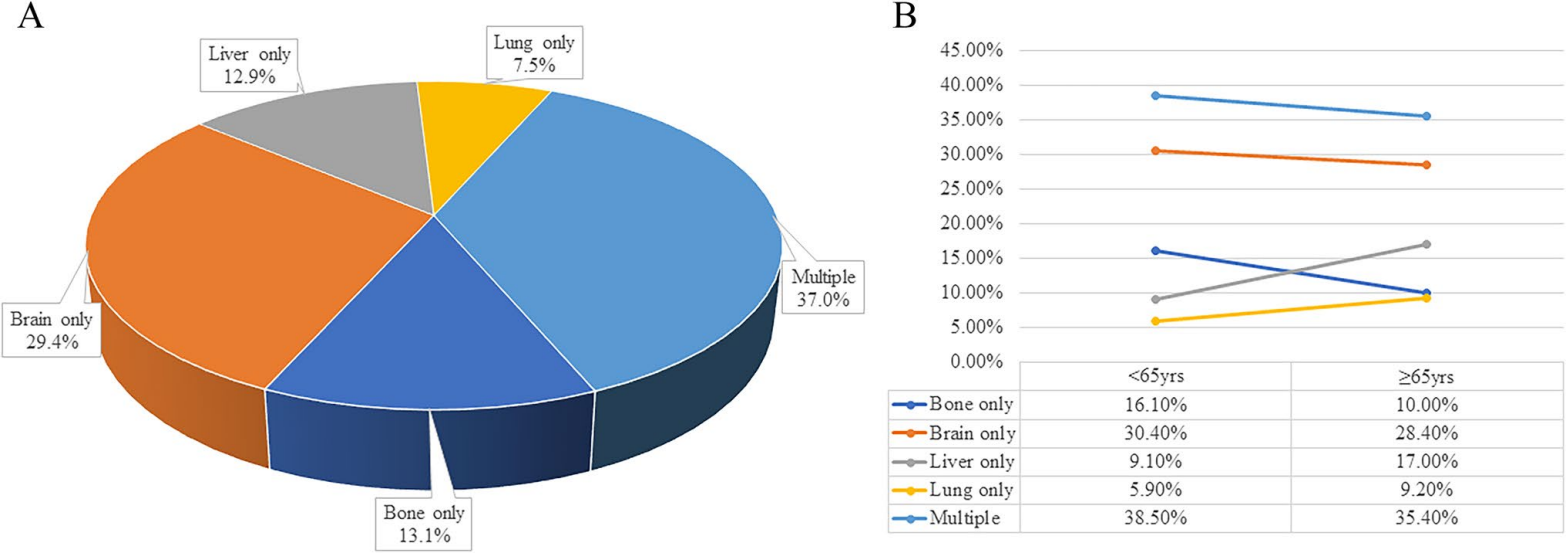
Metastasis pattern of large cell neuroendocrine carcinoma. Different metastasis sites for all patients (**A**) and patients of different age groups (**B**)

Patients experienced isolated lung metastases, isolated brain metastases, isolated liver metastases, isolated bone metastases and multiorgan metastases (MOM) were 7.5%, 29.4%, 12.9%, 13.1% and 37%, respectively (Fig. 1A). The incidence of liver metastasis (17% vs. 9.1%) and lung metastasis (9.2% vs. 5.9%) in the older group was significantly higher than that in the younger group (Fig. 1B). Patients with different metastatic sites were all more likely to receive chemotherapy, of which isolated lung metastases, isolated brain metastases, isolated liver metastases, isolated bone metastases and MOM accounting for 73.8%, 61%,65.3%,74% and 71.8%, respectively. Nevertheless, only a small percentage of patients underwent surgery and radiotherapy.

### Survival analysis

**Fig. 2:**
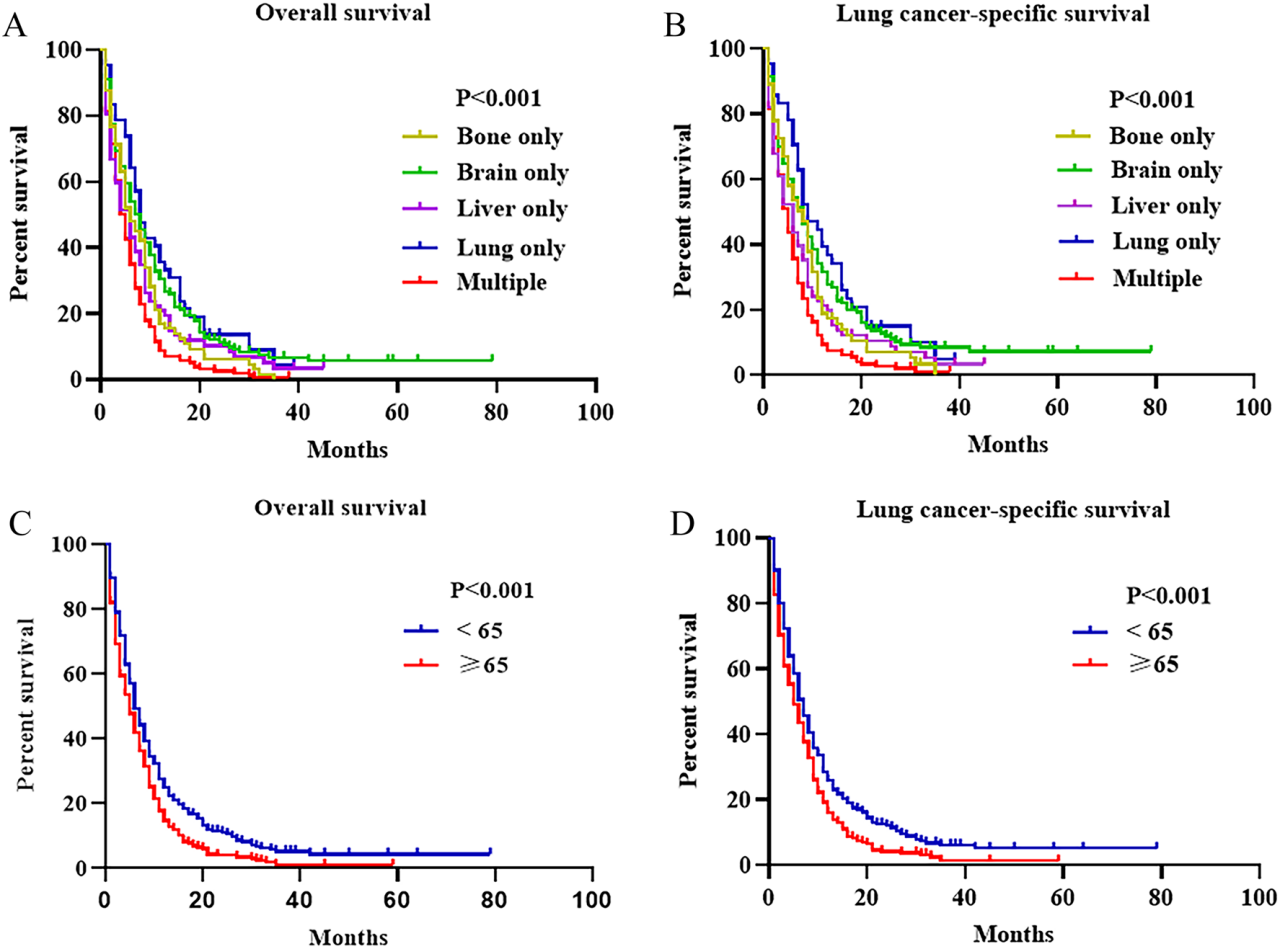
Kaplan–Meier curve of patients with advanced LCNEC. (**A**) OS (*P* < 0.001) and (**B**) LCSS (*P* < 0.001) of different metastasis pattern. (**C**) OS (*P* < 0.001) and (**D**) LCSS (*P* < 0.001) of different age groups. *OS* overall survival, *LCSS* lung cancer-specific survival

**Fig. 3:**
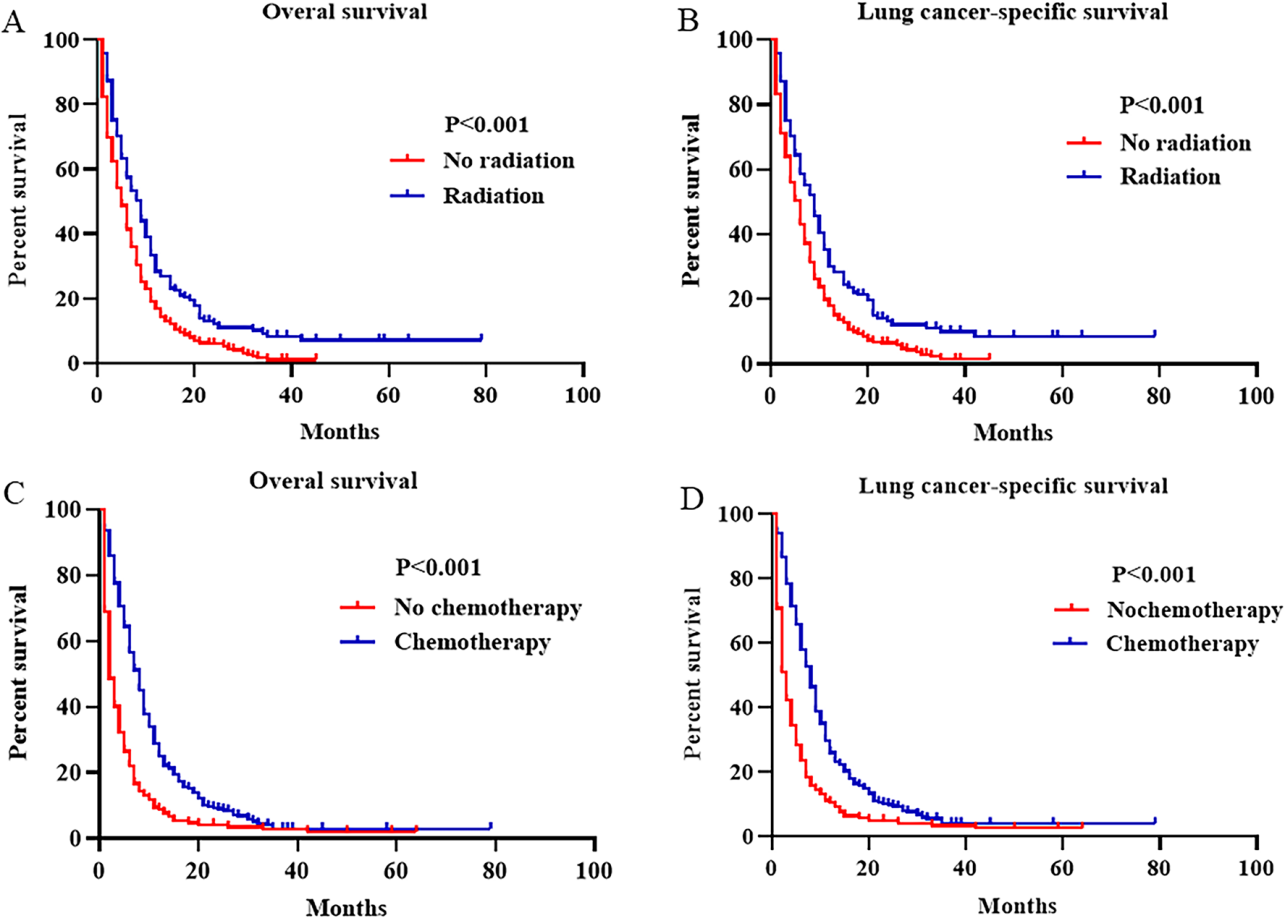
Effect of different treatment options on prognosis. Kaplan–Meier curve of patients with radiation for OS (**A**) and LCSS (**B**), and patients with chemotherapy for OS (**C**) and LCSS (**D**). *OS* overall survival, *LCSS* lung cancer-specific survival

The median survival time for the overall population was 6 months. The median survival time of lung metastases, brain metastases, liver metastases, bone metastases and MOM were 8, 6, 6, 7 and 4.5 months, respectively. Kaplan–Meier survival analysis demonstrated that the 2-year OS of lung metastases, brain metastases, liver metastases, bone metastases and MOM were 13.6%, 11.4%,10.3%,6.2% and 2.6%, respectively (Fig. 2A). Kaplan–Meier survival analysis demonstrated that the 2-year LCSS of lung metastases, brain metastases, liver metastases, bone metastases and MOM were 15%, 12.9%, 10.4%, 6.9% and 2.8%, respectively (Fig. 2B). Analysis of different age groups revealed that the median survival time was 5 months (IQR, 2–10) and 6 months (IQR, 3–12) in the older (≥ 65 years) and younger (< 65 years) groups, respectively (Fig. 2C, D). Patients aged ≥ 65 years had worse OS (HR: 1.247; 95% CI 1.039–1.496; *P* = 0.018) and LCSS (HR: 1.242; 95% CI 1.031–1.496: *P* = 0.022) than younger patients. Furthermore, patients received radiotherapy (HR: 0.716; 95% CI 0.565–0.906; *P* = 0.005) and chemotherapy (HR: 0.404; 95% CI 0.331–0.493; *P* < 0.001) were related to better OS (Fig. 3A, C), and the same phenomenon was observed in LCSS (Fig. 3B, D). Moreover, univariate Cox analysis indicated that race, T stage, N stage and metastatic site were also prognostic factors for OS and LCSS (Table2). However, in the adjusted multivariate Cox analysis, seven indicators except surgery were all independent prognostic factors for LCNEC. Taking isolated lung metastases as reference, isolated liver metastases (HR: 1.528; 95% CI 1.017–2.297; *P* = 0.041), isolated bone metastases (HR: 1.646; 95% CI 1.098–2.469; *P* = 0.016), and MOM (HR: 2.020; 95% CI 1.413–2.888; *P* < 0.001) as risk factors for OS, which were similar to the outcomes observed in LCSS (Table 3).

**Table 2:** Univariate analysis of OS and LCSS for patients with metastatic large cell neuroendocrine carcinoma

Characteristics	OS		LCSS	
	HR (95% CI)	*P* value	HR (95% CI)	*P* value
**Age**				
< 65	Reference		Reference	
≥ 65	1.353 (1.139–1.608)	**0.001**	1.345 (1.129–1.604)	**0.001**
**Sex**				
Female	Reference		Reference	
Male	1.090 (0.916–1.297)	0.332	1.048 (0.878–1.251)	0.605
**Race**				
White	Reference	0.080	Reference	**0.043**
Black	0.756 (0.577–0.991)	**0.043**	0.732 (0.554–0.969)	**0.029**
Other	0.770 (0.486–1.220)	0.265	0.711 (0.438–1.155)	0.168
**Marital status**				
Married	Reference	0.512	Reference	0.599
Unmarried	1.019 (0.595–1.743)	0.946	1.089 (0.912–1.301)	0.346
Unknown	1.127 (0.657–1.933)	0.665	0.937 (0.537–1.635)	0.818
**Laterality**				
Left	Reference	0.121	Reference	0.119
Right	1.017 (0.849–1.219)	0.853	1.014 (0.843–1.219)	0.885
Others	0.697 (0.481–1.011)	0.057	0.687 (0.468–1.007)	0.054
**Primary location**				
Upper lobe, lung	Reference	0.708	Reference	0.692
Middle lobe, lung	1.013 (0.676–1.518)	0.949	0.997 (0.660–1.505)	0.988
Lower lobe, lung	1.060 (0.856–1.312)	0.593	1.017 (0.817–1.265)	0.882
Main bronchus	1.158 (0.834–1.606)	0.381	1.127 (0.806–1.576)	0.484
Overlapping lesion of lung	1.801 (0.670–4.844)	0.244	1.836 (0.683–4.940)	0.229
Lung, NOS	0.927 (0.718–1.197)	0.561	0.890 (0.684–1.158)	0.386
**Grade**				
I-III	Reference	0.469	Reference	0.642
IV	1.212 (0.858–1.711)	0.275	1.100 (0.766–1.580)	0.607
Unknown	1.109 (0.904–1.359)	0.321	1.103 (0.897–1.357)	0.351
**T stage**				
T0-1	Reference	**0.008**	Reference	**0.004**
T2	1.311 (0.975–1.762)	0.073	1.365 (1.006–1.851)	**0.046**
T3	1.590 (1.176–2.152)	**0.003**	1.646 (1.205–2.247)	**0.002**
T4	1.630 (1.224–2.170)	**0.001**	1.710 (1.273–2.297)	** < 0.001**
Tx	1.305 (0.922–1.848)	0.133	1.312 (0.915–1.882)	0.140
**N stage**				
N0	Reference	**0.017**	Reference	**0.009**
N1	1.237 (0.886–1.727)	0.211	1.297 (0.924–1.822)	0.133
N2	1.424 (1.134–1.788)	**0.002**	1.471 (1.164–1.861)	**0.001**
N3	1.468 (1.123–1.919)	**0.005**	1.544 (1.173–2.030)	**0.002**
Nx	1.657 (1.026–2.676)	**0.039**	1.688 (1.031–2.762)	**0.037**
**Surgery**				
No/unknown	Reference		Reference	
Yes	0.583 (0.393–0.866)	**0.007**	0.584 (0.391–0.875)	**0.009**
**Radiation**				
No	Reference		Reference	
Yes	0.640 (0.523–0.783)	** < 0.001**	0.637 (0.518–0.783)	** < 0.001**
**Chemotherapy**				
No/unknown	Reference		Reference	
Yes	0.497 (0.412–0.598)	** < 0.001**	0.499 (0.413–0.604)	** < 0.001**
**Metastasis site**				
Lung Only	Reference	** < 0.001**	Reference	** < 0.001**
Brain Only	1.123 (0.786–1.604)	0.524	1.173 (0.810–1.698)	0.398
Liver Only	1.456 (0.978–2.166)	0.064	1.562 (1.037–2.352)	**0.033**
Bone Only	1.443 (0.972–2.141)	0.069	1.434 (0.950–2.164)	0.086
Multiple	1.949 (1.373–2.765)	** < 0.001**	2.063 (1.435–2.965)	** < 0.001**

*OS* overall survival, *LCSS* lung cancer-specific survival, *HR* hazard ratio, *CI* confidence interval

*P* values in bold signify a statistically significant difference

**Table 3:** Multivariate analysis of OS and LCSS for patients with metastatic large cell neuroendocrine carcinoma

Characteristics	OS		LCSS	
	HR (95% CI)	*P* value	HR (95% CI)	*P* value
**Age**				
< 65	Reference		Reference	
≥ 65	1.247 (1.039–1.496)	**0.018**	1.242 (1.031–1.496)	**0.022**
**Race**				
White		0.091		0.051
Black	0.752 (0.568–0.996)	**0.047**	0.729 (0.545–0.973)	**0.032**
Other	0.784 (0.491–1.250)	0.306	0.727 (0.444–1.189)	0.204
**T stage**				
T0-1		**0.018**		**0.009**
T2	1.250 (0.923–1.692)	0.149	0.288 (0.943–1.760)	0.112
T3	1.496 (1.094–2.045)	**0.012**	1.537 (1.113–2.122)	**0.009**
T4	1.612 (1.190–2.184)	**0.002**	1.686 (1.233–2.305)	**0.001**
Tx	1.184 (0.824–1.702)	0.360	1.174 (0.807–1.709)	0.401
**N stage**				
N0		**0.008**		**0.004**
N1	1.093 (0.773–1.545)	0.616	1.139 (0.800–1.620)	0.470
N2	1.519 (1.186–1.946)	**0.001**	1.573 (1.219–2.031)	**0.001**
N3	1.517 (1.137–2.022)	**0.005**	1.600 (1.192–2.148)	**0.002**
Nx	1.509 (0.911–2.500)	0.110	1.566 (0.933–2.628)	0.089
**Surgery**				
No/unknown	Reference		Reference	
Yes	0.881 (0.575–1.350)	0.559	0.889 (0.575–1.374)	0.596
**Radiation**				
No	Reference		Reference	
Yes	0.716 (0.565–0.906)	**0.005**	0.716 (0.563–0.911)	**0.007**
**Chemotherapy**				
No/unknown	Reference		Reference	
Yes	0.404 (0.331–0.493)	** < 0.001**	0.403 (0.329–0.495)	** < 0.001**
**Metastasis site**				
Lung Only	Reference	** < 0.001**	Reference	** < 0.001**
Brain Only	1.369 (0.931–2.014)	0.111	1.462 (0.981–2.178)	0.062
Liver Only	1.528 (1.017–2.297)	**0.041**	1.666 (1.095–2.534)	**0.017**
Bone Only	1.646 (1.098–2.469)	**0.016**	1.650 (1.083–2.516)	**0.020**
Multiple	2.020 (1.413–2.888)	** < 0.001**	2.144 (1.480–3.104)	** < 0.001**

*OS* overall survival, *LCSS* lung cancer-specific survival, *HR* hazard ratio, *CI* confidence interval

*P* values in bold signify a statistically significant difference

### Calibration and performance testing of nomogram

**Fig. 4:**
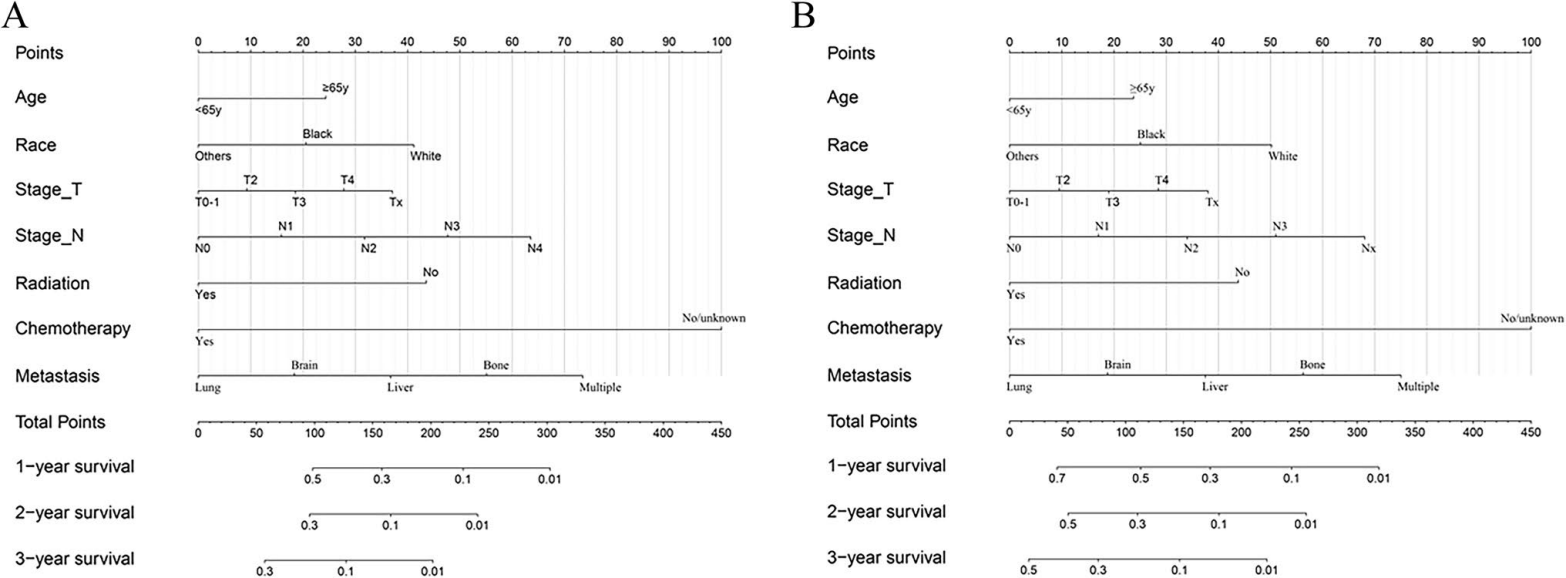
Predictive nomograms for predicting 1-, 2-, and 3-year OS and LCSS rate in patients with metastasis LCNEC. (**A**) OS rate; (**B**) LCSS rate. *OS* overall survival, *LCSS* lung cancer-specific survival

**Fig. 5:**
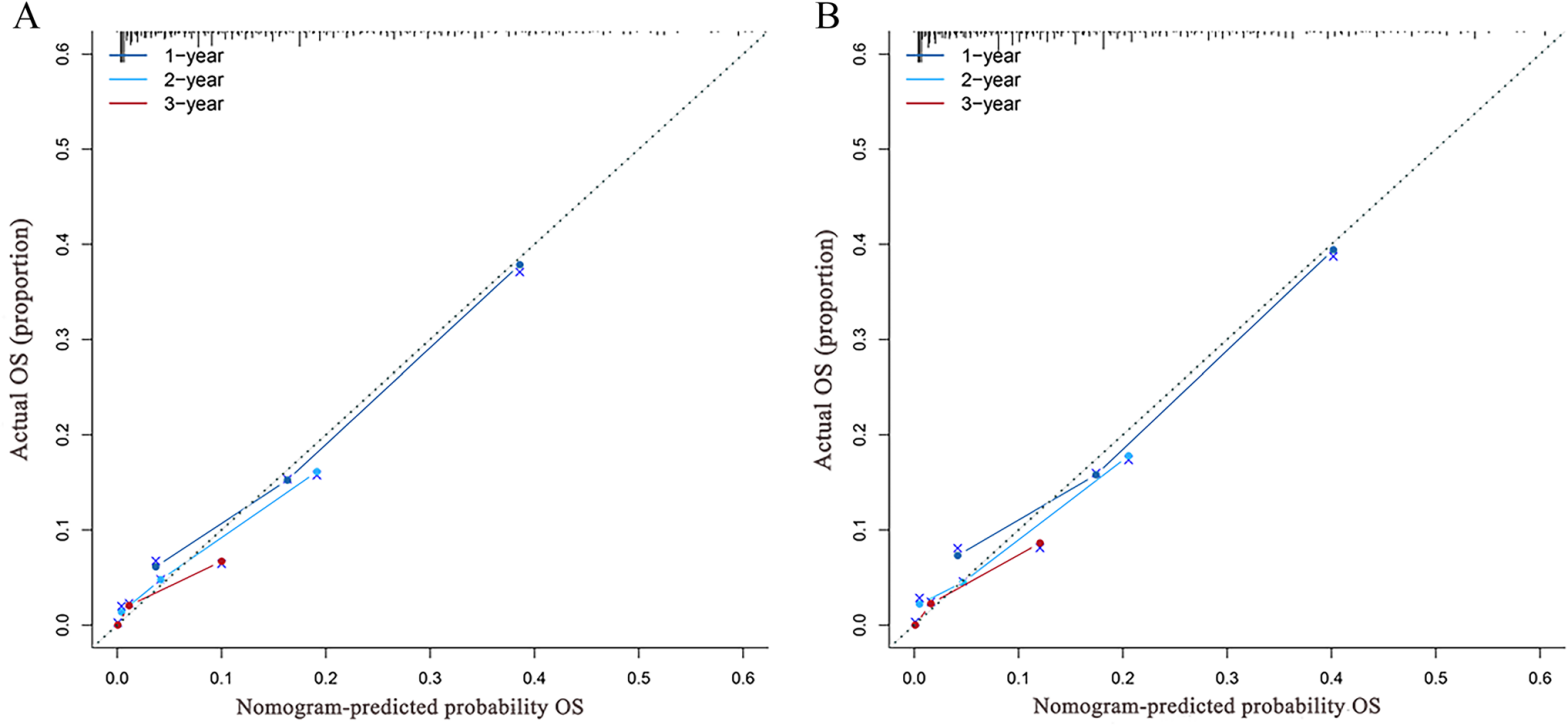
Calibration curves of the nomograms for predicting 1-, 2-, and 3‐year OS (**A**) and LCSS (**B**). *OS* overall survival, *LCSS* lung cancer-specific survival

Nomogram was developed based on independent prognostic factors identified by multivariate Cox regression analysis to predict 1-, 2- and 3-year OS (Fig. 4A) and LCSS (Fig. 4B). Additionally, chemotherapy and metastatic site had the widest scope of risk scores, which indicated the most pronounced effect on prognosis. The C-index values of nomograms were 0.692 (95% CI 0.665–0.719) for OS (Fig. 5A) and 0.693 (95% CI 0.666–0.720) for LCSS (Fig. 5B), which were considerably higher than the TNM staging system. Simultaneously, compared with the TNM staging model, the DCA curves showed excellent net benefit of the novel nomogram in predicting 1-, 2-, and 3-year OS (*Figure S2A-C*) and LCSS (*Figure S2D-F*).

## Discussion

In this large population-based retrospective research, we conducted the analysis of different metastasis patterns and developed comprehensive prognostic models for better predicting prognosis in advanced LCNEC for the first time. LCNEC is a rare form of lung cancer with an incidence of 3% and the incidence increasing (Fasano et al. 2015; Travis et al. 1991, 2021). There are some similarities between LCNEC and SCLC, both of them have high rate of invasiveness and distant metastasis. LCNEC is usually associated with male, age (median 65 years) and smoking habits and has poor OS and LCSS, which are similar to SCLC (Cos and Escuín 2014). Nevertheless, limited information is available on the clinical characteristics and treatment of patients with advanced LCNEC. Naidoo (2016) retrospectively analyzed 49 patients with stage IV LCNEC and revealed that the median age was 64 years, male and brain metastases were the most common. In this research, we extracted the data of 540 patients from the SEER database. Consistent with previous studies, the majority of patients were male with a median age of 64 years. Furthermore, a higher proportion of patients were white (84.7%) and the lesions tended to be located in the right lung (47.4%). The primary tumors in patients with different metastatic patterns were all mostly found in the upper lobe. And between different age groups, patients all frequently developed MOM (≥ 65 yrs, 28.4% and < 65 yrs, 30.4%) and among the isolated metastases, isolated brain metastases were predominant (≥ 65 yrs, 35.4% and < 65 yrs, 38.5%),which is in accordance with the higher rate of brain metastases in LCNEC reported by Naidoo et al. (2016) Several studies have confirmed that the brain is the most frequent site of distant metastasis in small cell lung cancer, which further supported the similarity in the biological behavior of these two types of tumors (Zou et al. 2021; Quan et al. 2004).

Then, we analyzed the impact of various factors on prognosis. The median survival for the overall population was 6 months, whereas in the research by Naidoo et al. (2016) for a cohort of 49 patients with stage IV LCNEC, the median survival was 10.2 months, which was significantly longer than in our study. This discrepancy may be due to the large difference in the base of the included population. Tumor metastasis destroying the function of the corresponding organ is the main cause of mortality in almost all solid tumors, which should be given sufficient attention (Dammert et al. 2019; Gupta and Massagué 2006).In this research, we analyzed the prognostic impact of different metastatic sites. The median survival time was significantly longer for isolated lung metastases (8 months) than that for MOM (4.5 months) (*P* < 0.05), and there were higher 2-year OS (13.6% vs 2.6%, *P* < 0.05) and LCSS (15% vs 2.8%, *P* < 0.05) relative to MOM. Additionally, our study reveals that age, race, T stage, N stage, chemotherapy and radiotherapy were independent prognostic factors for IV LCNEC. Advanced age tends to be a risk factor for poorer prognosis in patients suffering from tumors (Chen et al. 2020; Fuhrman et al. 2021; Bernard et al. 2020; Tan et al. 2021; You et al. 2021), this may be due to the fact that young and old patients experience different physiological changes related to comorbidities, immune status and nutritional status. Moreover, elderly patients have reduced renal and hepatic reserve function so that the potential for drug interactions and treatment-related toxicity are also increased. Similarly, patients aged > 65 years had worse OS and LCSS compared to younger patients in our study. Accordingly, age should be a valuable indicator for treatment consideration.

As a subtype of NSCLC, the significant role of surgery in early LCNEC has been demonstrated in several studies (Ma et al. 2021; Jiang et al. 2019; Peng et al. 2021; Yang et al. 2019). In advanced LCNEC, we found that surgery, although improving patient survival, has not yet been able to serve as an independent prognostic factor, which may be influenced by other factors and more advanced patients are being deprived of surgical opportunities. Therefore, in advanced LCNEC, a combination of multiple clinicopathological factors is needed to make treatment decisions. Consistent with conventional wisdom, our study showed that patients with advanced LCNEC were predominantly treated with chemotherapy and radiotherapy, and patients receiving these treatments showed significant survival advantages which could serve as independent OS and LCSS prognostic factors (Naidoo et al. 2016). Finally, the C-index of the nomogram was significantly higher than that of the AJCC staging model, indicating that the nomogram had more accurate and reliable predictive performance. The DCAs showed that the newly established nomogram has a higher net benefit and clinical application compared to the AJCC staging model.

This research was subject to some limitations. First, this was a retrospective research and patients with incomplete information were excluded from our study, which may lead to inherent and selection bias. Second, the SEER database does not include data on smoking history, comorbidities and functional status, which are often essential information affecting treatment decisions and disease prognosis. Finally, in recent years, targeted therapy and immunotherapy have greatly improved disease prognosis, however, above information are not included in the SEER database, which may reduce the predictive accuracy of the nomogram to some extent. A prospective study will be conducted to obtain data from Chinese patients to validate and optimize the model in the next step.

## Conclusions

we analyzed the metastasis patterns and established a novel, comprehensive prognostic model of advanced LCNEC based on a large population-based study for the first time. The model demonstrated excellent predictive performance and net benefit compared to traditional AJCC staging system, which could assist clinicians in guiding personalized treatment decisions and reduce the healthcare burden to some extent.

## Supplementary Information

Below is the link to the electronic supplementary material.

Supplementary file1 (PDF 321 KB)

## Data Availability

The original contributions presented in the study are included in the article/Supplementary Materials; further inquiries can be directed to the corresponding author.

## Abbreviations

LCNEC: Large cell neuroendocrine carcinoma
OS: Overall survival
LCSS: Lung cancer-specific survival
HR: Hazard ratio
CI: Confidence interval
DCAs: Decision-curve analyses
