# Supplementary material for: Metastasis pattern and prognosis of large cell neuroendocrine carcinoma: a population-based study
Source: J Cancer Res Clin Oncol. 2023 Jul 27;149(15):13511–21. doi: 10.1007/s00432-023-04975-w (PMC10590330; doi:10.1007/s00432-023-04975-w)

Figure S1 The flow diagram of eligible patients in this research.

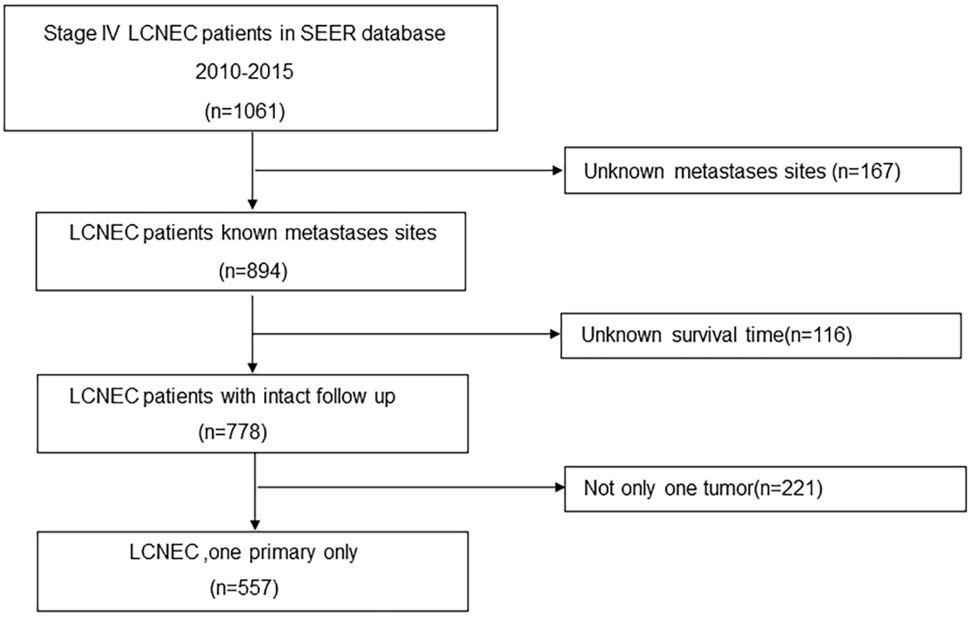

Figure S2 Decision curve of the nomograms and 7th edition AJCC-TNM staging system for predicting 1-, 2-, and 3-year OS (A-C) and LCSS (D-F). OS: overall survival; LCSS: lung cancer-specific survival.

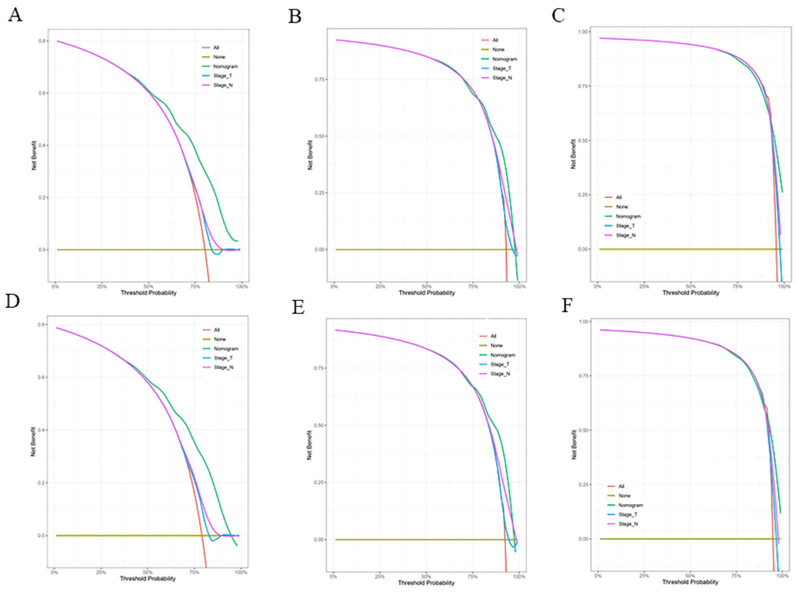

Supplement: Supplementary file 1 — Supplementary file1 (PDF 321 KB) [file 432_2023_4975_MOESM1_ESM.pdf]
